# Constructing Ecological Networks Based on Habitat Quality Assessment: A Case Study of Changzhou, China

**DOI:** 10.1038/srep46073

**Published:** 2017-04-10

**Authors:** Yu Gao, Lei Ma, Jiaxun Liu, Zhuzhou Zhuang, Qiuhao Huang, Manchun Li

**Affiliations:** 1 Jiangsu Provincial Key Laboratory of Geographic Information Science and Technology, Nanjing University, Nanjing 210023, China; 2 School of Geographic and oceanographic sciences, Nanjing University, Nanjing 210023, China; 3 Collaborative Innovation Center for the South Sea Studies, Nanjing University, Nanjing 210023, China

## Abstract

Fragmentation and reduced continuity of habitat patches threaten the environment and biodiversity. Recently, ecological networks are increasingly attracting the attention of researchers as they provide fundamental frameworks for environmental protection. This study suggests a set of procedures to construct an ecological network. First, we proposed a method to construct a landscape resistance surface based on the assessment of habitat quality. Second, to analyze the effect of the resistance surface on corridor simulations, we used three methods to construct resistance surfaces: (1) the method proposed in this paper, (2) the entropy coefficient method, and (3) the expert scoring method. Then, we integrated habitat patches and resistance surfaces to identify potential corridors using graph theory. These procedures were tested in Changzhou, China. Comparing the outputs of using different resistance surfaces demonstrated that: (1) different landscape resistance surfaces contribute to how corridors are identified, but only slightly affect the assessment of the importance of habitat patches and potential corridors; (2) the resistance surface, which is constructed based on habitat quality, is more applicable to corridor simulations; and (3) the assessment of the importance of habitat patches is fundamental for ecological network optimization in the conservation of critical habitat patches and corridors.

Ecosystems support life on Earth, and thus play a vital role in human well-being, either directly or indirectly^1^. In recent years, anthropogenic activity has facilitated the invasion of ecosystems by nonnative species and natural hazards, leading to the worsening of various environmental problems, including the degeneration of ecosystem services and a sharp decline in biodiversity^1^,^2^. Low continuity between habitat patches caused by the fragmentation of ecological landscapes (i.e., natural or semi-natural habitats) represents the greatest threat to biodiversity conservation^3^,^4^,^5^. However, growing environmental awareness and an improved understanding of how human communities interact with their environment have led to growing concerns about enhancing habitat patch continuity within ecosystems^6^,^7^. Nevertheless, recent studies show that economic growth has actually made humans more dependent on ecosystem services and biodiversity^8^. Therefore, it is particularly important to maximize ecosystem service values by constructing networks that enhance the functionality of urban ecosystem services^9^,^10^.

Research on ecological network construction has been widely carried out on a global scale^11^,^12^. The ecological network is a representation of the biotic interactions in an ecosystem, in which ecological corridors link protected habitat patches^13^. A habitat patch is a set of landscape patches, while a landscape patch is the basic unit—a relatively homogenous mosaic of familiar land-use types that differ from the surrounding background—that formulates landscape patterns. Previous studies have suggested that habitat patches are areas where organisms aggregate, representing stepping stones for migration^14^,^15^, while ecological corridors are narrow bands of vegetation that promote biological migration between the two habitat patches, allowing wild animals to survive^16^. These two landscape types form the core of ecological networks. However, one of the main limitations of studies on ecological newtorks is that the movement data of target speices are frequently unavailable. Therefore, there have been an increasing number of simulation-based studies on migration and biodiversity conservation in an attempt to address such defects^17^,^18^,^19^,^20^,^21^,^22^. In particular, methods based on graph theory emphasize the functional connection between habitat patches, which is the effective relationship between components of an ecological object or process with corresponding characteristic scales^16^. Graph theory has been gradually introduced to conduct research on the ecological networks of various land-use types, including cities, farmlands, and forests^20^,^23^,^24^,^25^.

The most important step when using graph theory is the construction of resistance surfaces. The resistance surface is a raster map (mosaic with a value, a larger value corresponds to higher resistance) indicating the level of disturbance or degree of difficulty that target species are expected to encounter when moving between patches, which significantly affects the outputs of ecological network research^26^. Therefore, many studies construct resistance surfaces based on various methods, such as biological behavior resistance estimates, expert scoring, entropy weighing, and landscape development intensity indexing^10^,^27^,^28^,^29^. However, most studies have constructed landscape resistance surfaces based on expertise and overall ratings for certain land-use types, leading to the resulting landscape resistance surfaces being heavily dependent on grading factors^5^,^26^. In fact, there are differences between the same land-use types owing to their different locations and surroundings. As a result, previous studies have weakened the differences in resistance of the same land-use type.

In this study, we propose a habitat quality-based method that involves simulating the sensitivity of different land-use types to impact factors. Within this framework, we evaluated the habitat quality and functionality of ecosystem services for each assessment unit^1^. Pixel-scale habitat quality was used to characterize the optimal survival, reproduction, and energy flow conditions that an assessment unit provided to organisms. Besides facilitating studies on how human activities affect habitat quality and animal migration, this information is expected to help estimate the landscape resistance of different assessment units. Good habitat quality promotes the dispersal of animals, and thus corresponds to low resistance. Although several studies on urban ecological network construction do exist^27^,^30^,^31^, a limited number of studies have analyzed the effects of different resistance surfaces on corridor simulations. In addition, these investigations weaken the significance assessments of habitat patches and corridors, which may be not conducive to the implementation of actual protection measures.

The ecological protection planning of Changzhou, China—which contains habitat patches but no corridors—was implemented to improve ecosystem services and biodiversity. Thus, this study proposes a set of procedures that could be applied to ecological network research by identifying potential corridors in Changzhou. Based on landscape ecology, graph theory, and habitat quality assessments, this study aimed to (1) propose a method for constructing landscape resistance surfaces; (2) quantitatively characterize potential corridors in the study area by using a minimum cumulative resistance model; (3) evaluate the importance of habitat patches and corridors; and (4) compare the effects of different methods of constructing resistance surfaces on potential corridor simulations and conduct an importance assessment of habitat patches and corridors. This information is expected to provide quantitative scientific data to enhance environmental protection in Changzhou.

## Study Area and Data Preparation

### Overview of the study area

Changzhou (119°08′–120°12′E, 31°09′–32°04′N) is located in the Taihu Lake Plain, Yangtze River Delta, in the southeastern part of Jiangsu Province, China. It has a subtropical monsoon climate, with an annual average temperature of 16.5 °C and an annual precipitation of 1063.71 mm. From 2006 to 2014, the built-up area in the city increased by 25.68%, demonstrating clear urban expansion. However, the percentage of farmland, forest, grassland, and water body areas offering ecological services has decreased. The shrinkage of ecological land area, the fragmentation of habitats, and the obstruction of corridors are major contributors to the deterioration of ecosystem services in Changzhou, and present an increasing burden on environmental protection.

### Source of data

The following datasets were provided by the Land and Resources Bureau: land-use change vector data (shapes with an attribute of its land-use type) from 2006 to 2014, administrative divisions, vector data of roads, vector data of built-up land, and ecological protection planning containing a nature reserve vector map of Changzhou. The nature reserve vector map is a highly important part of ecological protection planning in Changzhou. The map depicts the core area in Changzhou that could be protected by administrative means. Data regarding people per square kilometer of land area and Gross Domestic Product (GDP) per square kilometer in China (2010) were obtained from the Data Center for Resources and Environmental Sciences, Chinese Academy of Sciences (http://www.resdc.cn). Certain factors (such as land-use type, level of human disturbance, and landscape function) were used to classify the landscape types in Changzhou as farmland, forests, built-up land, riparian green space, and other types. Because the riparian green space is always a strip of land along a river or lake, we integrated riparian green space and water bodies as riparian green space. This information was used to generate the landscape classification map of Changzhou.

### Methodology

**Figure 1:**
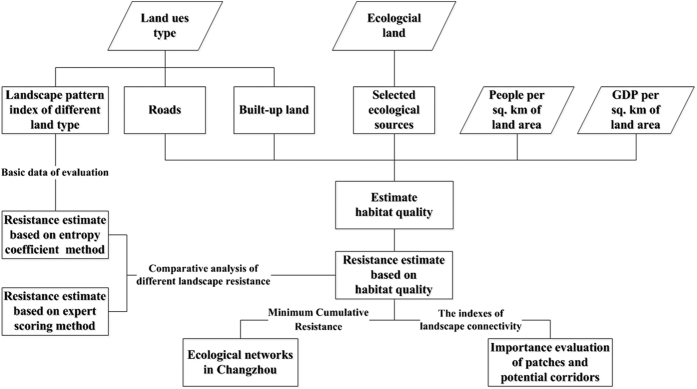
Technical flow chart.

We identified potential corridors based on graph theory. The three main steps were 1) the selection of habitat patches, 2) the construction of resistance surfaces, and 3) the identification of potential corridors. First, habitat patches were acquired from the nature reserve vector map. To analyze the effect of different resistance surfaces on corridors, we constructed resistance surfaces based on three different methods: (1) the habitat quality-based model, (2) the entropy coefficient method, and (3) the expert scoring method. Second, based on different resistance surfaces and the minimum cumulative resistance model, we identified all potential corridors in Changzhou. The results generated three groups of habitat patches and corridors reflecting the three methods we used. The resulting habitat patches, selected from the nature reserve vector map, were the same, while corridors differed as a result of differences in the resistance surfaces. Thus, we were able to compare the effects of the different methods of construction on the simulated potential corridors. Finally, the importance of both habitat patches and potential corridors were assessed. A flow chart of the techniques used is shown in Fig. 1.

### Target species and the selection of habitat patches

Connectivity is a species-dependent property^32^. Therefore, selecting target species that may be used as the basis for the study of ecological networks is necessary^27^. In this study, we focused on the common species of Changzhou, which were mostly small mammals, such as weasels, hares, and badgers. These animals prefer to move in vegetated habitats, such as forests and riparian green spaces. Connectivity is a species-dependent property^32^. Thus, based on the nature reserve vector maps provided by the Changzhou Bureau, we selected habitat patches that contained forested areas and riparian green spaces, and excluded river habitat patches, which would not suitable for our target species. These habitat patches serve as “sources” of matter, energy flow, and animal migration, and are also the origins where ecological processes develop^10^,^14^.

**Figure 2:**
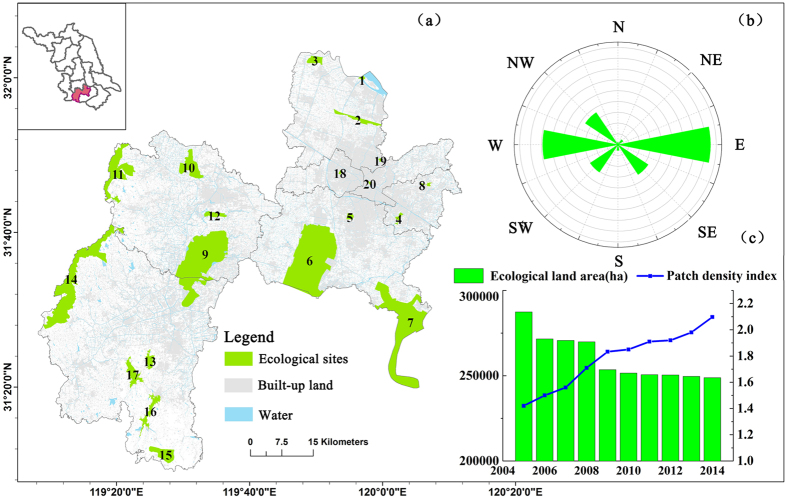
(**a**) Habitat patches in Changzhou, (**b**) differences in the spatial distribution of different habitat patches (where, based on the center of gravity of Changzhou, study area was divided into eight equal areas. Then, the area of habitat patches located in different sectors was statistically calculated), and (**c**) variations in ecological land area (the total area of farmland, forests, and water bodies in Changzhou) and the patch density index in Changzhou during 2006–2014 (where the left axis indicates the ecological land area and the right axis indicates the patch density index values). (Created by ArcMap, version 10.2, http://www.esri.com/. Boundaries of Jiangsu province and Changzhou, land-use type data and habitat patches acquired from Changzhou Land Resources Bureau).

Changzhou is one of the most developed cities in China. As a result, the habitat patches in this city are significantly affected by urbanization. Habitat patches are mostly distributed in the western and eastern parts of Changzhou, with fewer patches located in the north-northeastern part (Fig. 2(b)). The degree of ecological land fragmentation in Changzhou was evaluated using the patch density index^33^. The index grew from 1.42 in 2006 to 2.10 in 2012 (Fig. 2(c)), illustrating that habitat patches became increasingly fragmented. Thus, spatial fragmentation and uneven distribution lead to difficulties in constructing an effective ecological network. In this study, 20 ecological sources were selected, as shown in Table 1 and Fig. 2(a).

### Construction of landscape resistance surfaces

As shown in previous studies, the resistance surface has a major impact on the simulation of corridors^26^. Thus, to compare the effects of different landscape resistance surfaces on ecological network construction, and to verify the feasibility of applying the method proposed by this study landscape resistance, various methods used by previous studies were employed to construct different landscape resistance surfaces. Such methods included the entropy coefficient method^34^ and the expert scoring method^27^.

### Habitat quality-based method

Research has shown that habitat quality is associated with the intensity of human activity and the land-use types of surrounding areas^35^,^36^. The InVEST (Integrated Valuation of Ecosystem Service and Tradeoffs) model was developed jointly by Stanford University, the University of Minnesota, The Nature Conservancy, and the World Wildlife Fund^2^. This model simulates the combined effects of land-use changes and human activities on ecosystems, and it provides a visual tool that quantitatively evaluates ecosystem service functions^2^,^37^,^38^. The habitat quality component of the biodiversity module of this model was adopted in this study to analyze how the intensity of human activity influences habitat quality in Changzhou. The principles for calculating this model are:


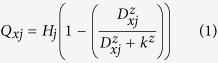


where *H*_*j*_ denotes the habitat suitability of land cover type *j*, 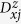 denotes the degree of habitat degeneration of land cover type *j* in raster *x, z* is a model parameter with a value of 2.5, and *k* is the half-saturation coefficient. During this process, five raster layers and two tables were compiled. First, we created a land-use map in raster format at a resolution of one pixel = 30 m × 30 m. Second, roads and built-up land were created in raster format at a resolution of one pixel = 30 m × 30 m to indicate obstructions to animal migration. Then, data regarding people per square kilometer of land area and GDP per square kilometer in Changzhou were included to indicate the intensity of human activity. Finally, attributes of threats, habitat types, and target species sensitivity to different threats were created as shown in Tables 2 and 3.

The habitat quality results produced by InVEST were presented as raster data in the range of 0 to 1. A larger value corresponds to better habitat quality, and vice versa. When InVEST is applied to the construction of landscape resistance surfaces, habitat quality is converted to landscape resistance. Units of better habitat quality have smaller landscape resistance, and vice versa. The equation governing conversion is:


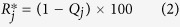


where *Q*_*j*_ is the result of habitat quality for raster unit *j*, and 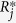 is the landscape resistance of the same unit. Within this process, the constructed resistance surface was completed based on habitat quality.

### Entropy coefficient method

**Figure 3:**
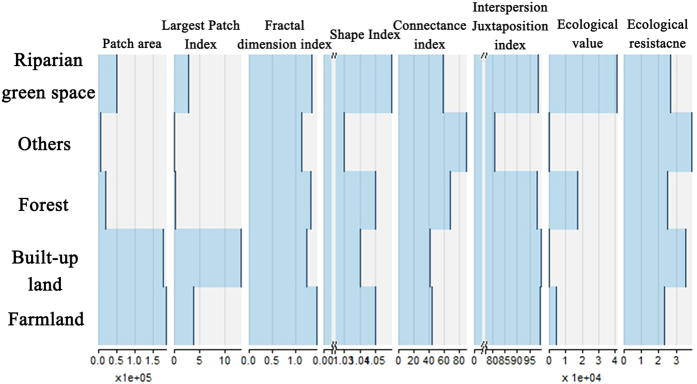
Landscape indexes of different land-use types (first seven columns from the left) and landscape resistance estimates (last column) used in the entropy coefficient method (the axis at the bottom of the figure is the value corresponding to the indexes shown at the top; the values of the indexes were calculated using Fragstats 4.2). (Created by Fragstats, version 4.2, http://www.umass.edu/landeco/research/fragstats/).

The entropy coefficient method is an objective weighting method in which weight is determined by entropy. Entropy is the amount of additional information needed to specify the exact physical state of a system. The greater the entropy, the more information it provides^34^. This process involves three main steps: 1) normalizing the initial information matrix, 2) calculating entropy weight, and 3) calculating resistance. In this study, landscape indexes and ecosystem service values were used as the ecological attributes for each land-use type in the weighted calculation (Fig. 3)^29^,^39^. Thus, the initial information matrix contains seven rows and five columns. To compare the different factors, the value of the factors was normalized by equations (3) and (4). *i* and *j* correspond to the number of rows and columns, respectively, in the initial information matrix. 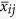 is the normalized result, *x*_*ij*_ is the original value, and 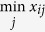 and 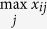 are the maximum and minimum value in *i*^th^ row, respectively.


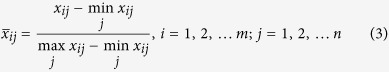



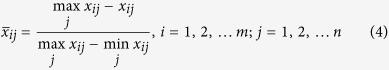


Through an integrated assessment of the volume of information carried by each factor, an integrated weight coefficient is calculated based on the abundance of information that a factor provides. The principles for the calculation are^39^:


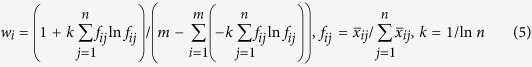


where *w*_*i*_ is the weighted coefficient of the corresponding factors. If *f*_*ij*_ = 0, we assume*f*_*ij*_ In *f*_*ij*_ = 0. Based on the normalized results of 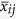 and *w*_*i*_, the final matrix could be calculated by 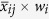. The resistance of different land-use types is defined as the sum of the columns in the final matrix.

### Expert scoring method

The expert scoring method examines the sensitivity of each landscape to human disturbance, and provides a quantitative characterization of the magnitude of its resistance^27^,^40^. First, we based the different land-use types on those suggested by experts of the selected focal species in Changzhou. Then, the resistance values were primarily chosen according to vegetation type, in addition to the area of green space and the degree of anthropogenic disturbance^30^. The resistance values were set between 1 and 1000 to indicate the level of disturbance or degree of difficulty that target species would encounter when moving between patches. The reference landscape resistance values are shown in Table 4.

### Simulation of potential corridors

Corridors are vital components of ecological networks. Corridors provide paths for animal migration, as well as conserving biodiversity and facilitating sustainable development^41^. As a result of increases in the intensity of human activity, ecological connectivity has sharply declined in Changzhou. In research on the construction of ecological networks, two corridor simulation methods are primarily used: (1) directly tracking animal migration paths and simulating ecological processes^42^, which is highly dependent on data availability, is difficult to perform, and is less commonly used in urban environment studies^26^; and (2) model simulation, which resolves the difficulties of data collection to some extent, resulting in this model being widely applied in ecological network research^27^. In particular, the minimum cumulative resistance (*MCR*) model is a raster-based optimization algorithm that was originally designed to find the least-expensive path for a link between two nodes, since we assumed that target species follow an optimum route to minimize exposure to intervening low-quality landscape patches. This model reveals obstructions and boosts underlying migration processes^19^. This model is often combined with landscape graph theory to reflect complex network structures and ecosystem-scale relationships between energy, matter, gene migration, and the underlying surface^43^. In this process, two layers are used. The first is the habitat patches layer (treated as the nodes). The second is the resistance surface layer. The resistance value for each potential corridor is determined by the number of raster units that are covered and the landscape resistance of each unit:


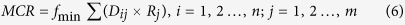


where *MCR* is the resistance of a potential corridor between two habitat patches, *D*_*ij*_ is the number of raster units between two habitat patches, *R*_*j*_ is the resistance of a raster type, *i* is the number of habitat patches, and *j* is the number of raster units on the resistance surfaces.

### Network continuity evaluation

Landscape continuity is the relationship between landscape elements in their spatial structures. This parameter is used to determine the structural characteristics of a landscape. It is commonly measured by continuity indexes, including the structural continuity index and the functional continuity index^44^. In recent years, quantitative assessments on continuity and the complexity of ecological networks based on landscape graph theory have increased^45^, with new indexes being developed, such as the overall network continuity index, based on graph theory^46^,^47^. Compared with traditional descriptive indexes on network complexity and continuity, graph theory-based evaluation indexes integrate the ecological attributes of a habitat into the calculation, giving these metrics ecological significance. Landscape continuity could be used to assess the importance of habitat patches and corridors. The evaluation indexes used in this study are shown in Table 5.

The results of habitat patch importance vary with the selection of different indexes. In this study, importance indexes *dIIC* and *Dpc* (Table 5) corresponded to the integral index of continuity (*IIC*) and probability of continuity (*PC*), respectively, and were applied to evaluate the significance of each habitat patch in upholding landscape continuity. Using the hierarchical clustering module in SPSS 21.0, each index was classified into three classes. As a result, the importance of habitat patches and corridors in the ecological network in Changzhou was determined. Thus, we determined the importance of habitat patches and corridors in the ecological network in Changzhou. At least one of the indexes ranked as “very important”, with such habitat patches being classified as a “primary patch”. When both indexes were ranked as “ordinary”, the habitat patch was considered to be a “tertiary patch.” In all other cases, habitat patches were considered to be a “secondary patch”. The same method was used to classify potential corridors.

## Results and Discussion

### Landscape resistance threshold recognition

Previous studies have made the assumption that ecological connection becomes effective when a potential corridor between habitat patches has a cumulative resistance that is smaller than the threshold^48^,^49^. Therefore, the threshold represents the highest cumulative resistance that limits the migration of species between two patches. The methods used to identify the threshold value can be divided into two parts. The first part tracks the maximum moving distance of the target species^49^. The second part uses landscape continuity indexes and statistical principles, catching the breaking point or stable value of the calculated index values at different thresholds^48^.

**Figure 4:**
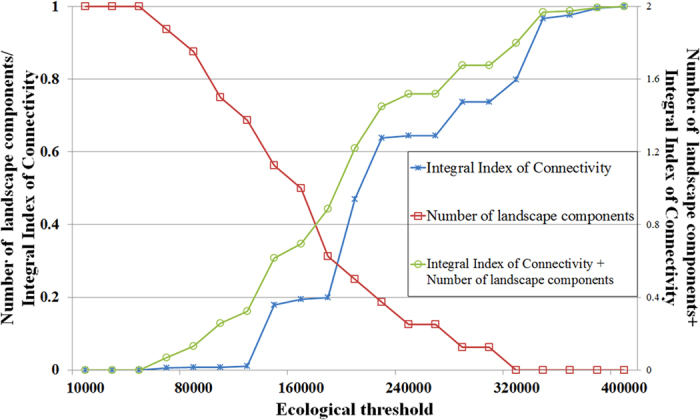
Variations in the integral index of continuity (IIC), and number of landscape components (NC) based on landscape cumulative resistance. The left vertical axis represents the normalized results of IIC and NC, while the right vertical axis is the sum of normalized IIC and normalized NC.

The second method was expected to be more suitable for the present study. All links between habitat patches were calculated using Linkage Mapper^50^ and Conefor Sensinode 2.6. Supporting previous studies, we found that calculating the threshold distance based on the highest number of landscape components (*NC*) is credible^48^. Thus, *IIC* and *NC* were used in this study. Two indexes were used to indicate connectivity between habitat patches. During this process, the values of *IIC* and *NC* can be calculated from a given hypothetical threshold. With a step defined as a landscape resistance value of 10000, the hypothetical threshold ranges from 0 to 400000, leading to variation in the index value. To compare the two indexes, the results of the calculation were normalized based on equation (3). For *NC*, a low value indicates good connectivity, while a high value indicates good connectivity for *IIC*. Thus, the green curve in Fig. 4 indicates the sum of these two indexes as calculated by:


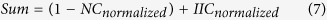


where *Sum* is the sum value of these two indexes, *NC*_*normalized*_ is the normalized result of *NC*, and *IIC*_*normalized*_ is the normalized result of *IIC*. Based on equation (7), we conclude that a high value of *Sum* indicates good connectivity.

The variation of *IIC*_*normalized*_ and *NC*_*normalized*_ showed that, when landscape resistance reaches 340000, *IIC* no longer increases rapidly, but becomes steady. In comparison, *NC* no longer varies, with the sum of these two indexes producing a steady curve (Fig. 4). Therefore, a landscape resistance threshold of 340000 was confirmed to be applicable to Changzhou, with any values exceeding this threshold signifying disconnection.

The probability of continuity (*PC*), which is used to assess the importance of habitat patches, indicates the probability of a species migrating between habitat patches. If the value calculated by the *PC* is relatively high (nearly 1), habitat patches might be connected by the potential corridors. To compare *IIC* with *PC*, a coherent setting for *IIC* and *PC* is needed, which is calculated by a decreasing negative exponential function of distance^51^. In this study, a *PC* exceeding the threshold must be small, and was therefore set as 0.05.

### Ecological network construction

**Figure 5:**
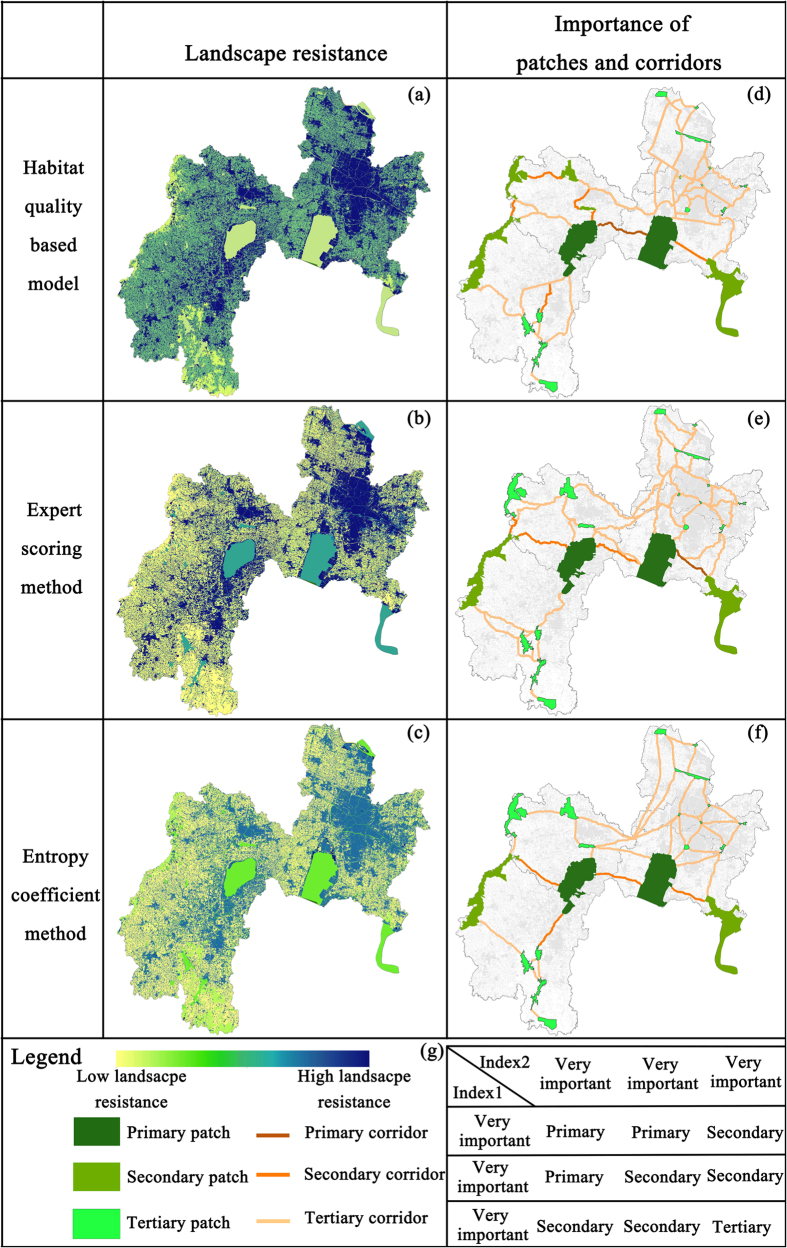
Landscape resistance estimates based on (**a**) the habitat quality-based model, (**b**) the expert scoring method, and (**c**) the entropy coefficient method. Results of the potential corridor simulations, importance assessment of habitat patches, and potential corridors based on different models of resistance surfaces are presented according to (**d**) the habitat quality-based model (**e**) the entropy coefficient method, and (**f**) the expert scoring method. The legend corresponding to panels a–f is located at the bottom, where a yellow to blue scale indicates low to high resistance, respectively; the three types of patches are indicated by three shades of green; and the three types of corridors are indicated by light orange, orange, and red lines. (**g**) Principle of the importance evaluation of habitat patches. (Created by ArcMap, version 10.2, http://www.esri.com/).

An ecological network is the framework for environmental protection in a city. The identification of important habitat patches^9^ and the functional connection between them^5^,^10^ is critical for constructing ecological networks. The resistance surface covering the entire study area, together with the least-cost paths between pairs of habitat patches, is shown in Fig. 5. An ecological network consists of habitat patches and potential corridors. Spatially, habitat patches are composed of landscape patches along the path of minimum resistance^52^. The potential corridors are strips connecting habitat patches, of which the accumulated resistance is lowest between habitat patches of all potential connections. However, the width of corridors could vary in different environments^27^. In this study, we assumed that a corridor width of 30 m could accelerate migration, which represented the cell size of the resistance surface.

### Effects of landscape resistance surfaces

To compare the landscape resistance surfaces that were constructed, the average habitat quality of different landscapes was normalized, as illustrated in Table 6. The estimated resistance was smaller for forests, farmlands, and riparian green spaces, which are less disturbed, highly vegetated, and more favorable for animal migration. In contrast, clear obstructions to animal migration were identified for roads and built-up land. Nevertheless, as a result of rapid urban expansion and the degeneration of the habitat quality of farmlands in Changzhou, the habitat quality-based resistance estimate for farmlands was higher than the estimates obtained by the other two methods. The results showed that while different resistance surfaces led to significant spatial offsets in the simulation of potential corridors, these results demonstrated high consistency in the identification of important habitat patches and corridors (Fig. 5). Concerning assessment units, we found that the model constructed in our study is more sophisticated than the other two models, and is more suitable for research on ecological networks in fragmented landscapes^1^,^2^.

In addition, we analyzed the composition of potential corridors, which also revealed the effect of landscape resistance surfaces. We found that the target species preferred to move in vegetative habitats and riparian green spaces. However, different landscape resistance surfaces generated different corridor compositions. For instance, the corridors that were simulated from resistance surfaces constructed by the habitat quality-based model (Table 6) consisted of 3.45% built-up land. This percentage was lower than those obtained by the expert scoring method and the entropy coefficient method (6.27% and 16.04%, respectively). As the main potential corridors are riparian green spaces, followed by forest and farmland, the results indicate that the resistance surfaces constructed by the habitat quality-based model are more suitable for ecological network analyses in Changzhou. Hence, under conditions with insufficiently detailed ecological data, or when it is difficult to obtain such data through experiments, it is more feasible to carry out ecological network analyses using the habitat quality-based model and the minimum cumulative resistance (*MCR*) model, rather than other methods, such as mark and capture^53^ and wireless wildlife tracking^5^.

### Importance of habitat patches

Once the ecological corridors are defined, the assessment of habitat patch importance is fundamental for optimizing the ecological network to construct and conserve critical patches and corridors^27^. In Changzhou, the Ge Lake and Changdang Lake Wetland Conservation Areas ranked high when evaluating the importance of habitat patches (Fig. 5), as these areas contain extensive habitat patches and are well connected to natural landscapes in the central and southern parts of Changzhou. Wawushan Provincial Park and the Xinlong Ecological Forest belong to linear habitat patches and form natural corridors (Fig. 5). These two sites reduce landscape resistance against animal migration, and are essential for enhancing the continuity of the ecological network. Nature Reserves in the central and southern parts of Changzhou, such as the Qianzi Lake Wetland Conservation Area, Yancheng Park, the Songjian Lake Wetland Park, and the Shahe Reservoir Restoration Area, were smaller than the Ge Lake and Taihu Lake Conservation Areas. However, these areas contain many potential corridors that could serve as excellent stepping-stone patches, which are advantageous to biological dispersal between habitat patches in regions with intensive human activity^54^. The inclusion of stepping-stone patches reduced the threats to biodiversity owing to isolated habitat patches, and also reduced resistance against animal migration. The results suggested that fragmented patches rarely provide favorable habitats for organisms to survive and reproduce, but clearly facilitate animal migration^30^.

### Importance of corridors

At present, the *IIC* of the ecological network in the study area is relatively low. The growth of *IIC* and resistance shows a logistic function (Fig. 4). When the resistance threshold was set as 340000, *IIC* became stable, with a value of just 0.007, signifying little potential for any connection between habitat patches. Among the potential corridors studied, the most important ecological connections were located between Taihu Lake and Ge Lake and between Ge Lake and the Changdang Lake Wetland Conservation Areas. The connections between Changdang Lake and the Qianzi Lake Conservation Areas and between Changdang Lake and the Liyang West Park were also of high importance. These four potential corridors combined with habitat patches to form a fundamental T-shaped habitat structure in Changzhou. This result is consistent with previous studies, which showed that rivers and coastal areas could be used as a means for ecological processes to enhance the connectivity of ecological networks and improve urban habitats in regions where ecological land is limited^5^.

## Conclusions

This study suggested the use of model simulations to construct ecological networks. An ecological network was constructed for the city by integrating habitat quality, graph theory, and the minimum cumulative resistance model. Our results verify the reliability of using this method in ecological network research. It was concluded that (1) the proposed landscape resistance surface method provides a way of effectively overcoming research limitations caused by insufficient experimental data; (2) the use of different methods in constructing resistance surfaces considerably affects the delineation of potential corridors, but only slightly influences the evaluation of the importance of habitat patches and potential corridors; (3) the suggested procedure is reliable because the potential corridors are mostly composed of green spaces rather than built-up land; and (4) habitat patches of high importance and good quality should be prioritized in regions with limited green space. However, this study did not consider how corridor width and complexity affect the migration of different species. To promote regional sustainable development, optimal corridor widths for species migration and multi-scale compound ecological networks should be determined by future studies.

## Additional Information

**How to cite this article**: Gao, Y. *et al*. Constructing Ecological Networks Based on Habitat Quality Assessment: A Case Study of Changzhou, China. *Sci. Rep.* **7**, 46073; doi: 10.1038/srep46073 (2017).

**Publisher's note:** Springer Nature remains neutral with regard to jurisdictional claims in published maps and institutional affiliations.

## Figures and Tables

**Table 1:** Selected ecological sites in Changzhou.

No.	Name	No.	Name	No.	Name
1	Yangtze River Water Quality Protection Zone	8	Hengshan Ecological Forest	15	Nanshan Water Conservation Area
2	Xinlong Ecological Forest	9	Changdang Lake Wetland	16	Shahe Reservoir Water Conservation Area
3	Xiaohuangshan Ecological Forest	10	The Waters of Lake Fisheries	17	Daxi Reservoir Water Conservation Area
4	Songjian Lake Wetland Park	11	Maoshan Water Conservation Area	18	Qingfeng Park
5	Yancheng Forest Park	12	Qianzi Lake Wetland Protection Area	19	Changzhou Dinosaur Park
6	Ge Lake Wetland	13	Western Suburbs of Liyang Forest Park	20	Hongmei Park
7	Taihu Lake Wetland Protection Area	14	Wawushan Provincial Park		

**Table 2:** Attributes of threats.

Threat	Maximum impact distance/km	Weight	Linear recession relation
Population	6.0	0.8	linear
Gross Domestic Product (GDP)	7.0	0.9	linear
Built-up land	5.0	0.7	linear
Roads	2.0	0.5	linear

**Table 3:** Habitat types and degree of sensitivity to each threat.

Land-use type ID LULC	Land-use type Name	Habitat Suitability Habitat	Population	GDP	Built-up land	Roads
1	Farmland	0.4	0.6	0.7	0.5	0.2
2	Forest	0.8	0.7	0.8	0.6	0.4
3	Riparian green space	0.9	0.9	0.9	0.9	0.6
4	Built-up land	0.0	0.0	0.0	0.0	0.0
5	Others	0.0	0.0	0.0	0.0	0.0

**Table 4:** Landscape resistance estimates based on the expert scoring method.

Land-use type	Range of landscape resistance
Farmland	10
Forest	50
Riparian green space	600
Built-up land	1000
Others	700

**Table 5:** Landscape continuity indexes^45^,^46^,^47^.

Indexes	Definition
Number of landscape components	A landscape component is the combination of habitat patches and corridors. Its habitat patches are connected by corridors. If one (or a group of) habitat patch (es) is not connected with others, then it is considered to be a single landscape component
Integral Index of Continuity 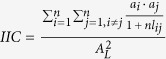	*n* denotes the total number of habitat patches; *a*_*i*_ and *a*_*j*_ denote the area of habitat patch *i* and that of habitat patch *j*; *nl*_*ij*_ denotes the number of connection between patches *i* and *j*; *A*_*L*_ is the total regional area
Probability of Continuity 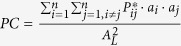	*n* denotes the total number of habitat patches; 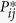 is the probability of directional migration between habitat patch *i* and habitat patch *j*; 0 < *PC* < 1
Importance Index (I_remove_) 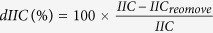 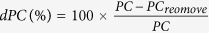	*IIC(PC*) denotes the continuity index; *IIC*_*remove*_ (*PC*_*remove*_) is the index after removing one habitat patch. Each iteration removes one habitat patch. Greater *DIIC(dPC*) indicates that a particular habitat patch is of higher importance in the network

**Table 6:** Normalized results of landscape resistance and the composition of corridors based on different resistance surfaces.

Land-use type	Riparian green space	Forest	Farmland	Others	Built-up land
Resistance	Normalized result of landscape resistance				
Habitat quality-based method	0.00	0.11	0.56	0.94	1.00
Entropy coefficient method	0.24	0.13	0.00	1.00	0.77
Expert scoring method	0.60	0.00	0.04	0.70	1.00
Composition of corridors					
Habitat quality-based method	89.79%	3.71%	3.03%	0.01%	3.45%
Entropy coefficient method	11.71%	1.31%	70.70%	0.24%	16.04%
Expert scoring method	3.21%	8.87%	81.58%	0.07%	6.28%

## References

[b1] Chiang L. C. . Simulation of ecosystem service responses to multiple disturbances from an earthquake and several typhoons. Landscape Urban Plan. 122, 41–55 (2014).

[b2] Terrado M. . Model development for the assessment of terrestrial and aquatic habitat quality in conservation planning. Sci. Total Environ. 540, 148–154 (2015). 10.1016/j.scitotenv.2015.03.064 25836757

[b3] Cook E. A. Landscape structure indices for assessing urban ecological networks. Landscape Urban Plan. 58, 269–280 (2002).

[b4] Pascual-Hortal L. & Saura S. Impact of spatial scale on the identification of critical habitat patches for the maintenance of landscape connectivity. Landscape Urban Plan. 83, 176–186 (2007).

[b5] Carvalho F., Carvalho R., Mira A. & Beja P. Assessing landscape functional connectivity in a forest carnivore using path selection functions. Landscape Ecol. 31, 1021–1036 (2016).

[b6] Fischer J. & Lindenmayer D. B. Landscape modification and habitat fragmentation: a synthesis. Global Ecol. Biogeogr. 16, 265–280 (2007).

[b7] Zetterberg A., Mörtberg U. M. & Balfors B. Making graph theory operational for landscape ecological assessments, planning, and design. Landscape Urban Plan. 95, 181–191 (2010).

[b8] Guo Z. W., Zhang L. & Li Y. Increased dependence of humans on ecosystem services and biodiversity. PLoS One. 5, e13113 (2010). 20957042 10.1371/journal.pone.0013113 PMC2948508

[b9] Xu W. W., Sun X., Zhu X. D., Zong Y. G. & Li Y. F. Recognition of important ecological nodes based on ecological networks analysis: A case study of urban district of Nanjing. Acta Ecologica Sinica. 32, 1264–1272 (in Chinese with English abstract) (2012).

[b10] Chen C. D., Meurk D. C., Ignatieva E. M., Stewart H. G. & Wu S. J. Identifying and evaluating functional connectivity for building urban ecological networks. Acta Ecologica Sinica. 35, 6414–6424 (in Chinese with English abstract) (2015).

[b11] Borrett S. R., Fath B. D. & Patten B. C. Functional integration of ecological networks through pathway proliferation. J Theor. Biol. 245, 98–111 (2007). 17084414 10.1016/j.jtbi.2006.09.024

[b12] Saura S. & Rubio L. A common currency for the different ways in which patches and links can contribute to habitat availability and connectivity in the landscape. Ecography. 33, 523–537 (2010).

[b13] Bennett G. & Mulongoy L. J. Review of experience with ecological networks, corridors and buffer zones. *Secretariat of the Convention on Biological Diversity, Montreal*. Technical Series 23 (2006).

[b14] Chen L., Fu B. & Zhao W. Source-sink landscape theory and its ecological significance. Frontiers of Biology in China 3, 131–136 (in Chinese with English abstract) (2008).

[b15] Hall L. S. & Morrison M. L. The habitat concept and a plea for standard terminology. Wildlife Soc. B. 25, 173–182 (1997).

[b16] Wu J. G. Landscape ecology-concepts and theories. Chinese Journal of Ecology 19, 42–45 (in Chinese with English abstract) (2000).

[b17] Elith J. & Leathwick J. R. Species distribution models: ecological explanation and prediction across space and time. Annu. Rev. Ecol. Evol. Syst. 40, 677–697 (2009).

[b18] Kuemmerlen M. . Integrating catchment properties in small scale species distribution models of stream macroinvertebrates. Ecol. Modell. 277, 77–86 (2014).

[b19] Adriaensen F. . The application of ‘least-cost’ modelling as a functional landscape model. Landscape Urban Plan. 64, 233–247 (2003).

[b20] Mcrae B. H., Dickson B. G., Keitt T. H. & Shah V. B. Using circuit theory to model connectivity in ecology, evolution, and conservation. Ecology. 89, 2712–2724 (2008). 18959309 10.1890/07-1861.1

[b21] Rudnick D. A. . The role of landscape connectivity in planning and implementing conservation and restoration priorities. Issues in Ecology. 16, 1–20 (2012).

[b22] Yang T., Jing D. & Wang S. Applying and exploring a new modeling approach of functional connectivity regarding ecological network: A case study on the dynamic lines of space syntax. Ecol. Modell. 318, 126–137 (2014).

[b23] Minor E. S. & Urban D. L. A graph-theory framework for evaluating landscape connectivity and conservation planning. Conserv. Biol. 22, 297–307 (2008). 18241238 10.1111/j.1523-1739.2007.00871.x

[b24] Minor E. S. & Urban D. L. Graph theory as a proxy for spatially explicit population models in conservation planning. Ecol. Appl. 17, 1771–82 (2008). 10.1890/06-1073.1 17913139

[b25] Urban D. L., Minor E. S., Treml E. A. & Schick R. S. Graph models of habitat mosaics. Ecol. Lett. 12, 260–273 (2009). 19161432 10.1111/j.1461-0248.2008.01271.x

[b26] Graves T., Chandler R. B., Royle J. A., Beier P. & Kendall K. C. Estimating landscape resistance to dispersal. Landscape Ecol. 29, 1201–1211 (2014).

[b27] Gurrutxaga M., Lozano P. J. & del Barrio G. GIS-based approach for incorporating the connectivity of ecological networks into regional planning. J. Nat. Conserv. 18, 318–326 (2010).

[b28] Pereira M., Segurado P. & Neves N. Using spatial network structure in landscape management and planning: A case study with pond turtles. Landscape Urban Plan. 100, 67–76 (2011).

[b29] Zhang L., Li S. U., Wang J. K. & Ming C. Establishment of ecological network based on landscape ecology in Anshan. Chinese Journal of Ecology 33, 1337–1343 (in Chinese with English abstract) (2014).

[b30] Kong F., Yin H., Nakagoshi N. & Zong Y. Urban green space network development for biodiversity conservation: Identification based on graph theory and gravity modeling. Landscape Urban Plan 95, 16–27 (2010).

[b31] Li H., Chen W. & He W. Planning of Green Space Ecological Network in Urban Areas: An Example of Nanchang, China. Int. J. Environ. Res. Public Health 12, 12889–12904 (2015). 26501298 10.3390/ijerph121012889 PMC4627006

[b32] Bani L., Baietto M., Bottoni L. & Massa R. The use of focal species in designing a habitat network for a lowland area of Lombardy, Italy. Conserv Biol, 16, 826–831 (2002).

[b33] Qiu F., Laliberté L., Swallow B. & Jeffrey S. Impacts of fragmentation and neighbor influences on farmland conversion: A case study of the Edmonton-Calgary Corridor, Canada. Land Use Policy. 48, 482–494 (2015).

[b34] Zhao B., Ren F., Yu H., Xu A. & Hao Y. Research on application of customer satisfaction index model—View_based on PLS and information entropy-weight method. *International Conference on E -Business and E -Government IEEE*, pp.1–4 (2011).

[b35] Wu J. S., Cao Q. W., Shi S. Q., Huang X. L. & Lu Z. Q. Spatio-temporal variability of habitat quality in beijing-tianjin-hebei area based on land use change. Chinese Journal of Applied Ecology 26, 3457–3466 (in Chinese with English abstract) (2015). 26915203

[b36] Romero-Calcerrada R. & Luque S. Habitat quality assessment using Weights-of-Evidence based GIS modelling: The case of Picoides tridactylus as species indicator of the biodiversity value of the Finnish forest. Ecol. Modell. 196, 62–76 (2006).

[b37] Goldstein J. H. . Integrating ecosystem-service tradeoffs into land-use decisions. Proc. Natl. Acad. Sci. USA 109, 7565–7570 (2012). 22529388 10.1073/pnas.1201040109 PMC3358905

[b38] Nelson E. . Projecting global land-use change and its effect on ecosystem service provision and biodiversity with simple models. PLoS One 5, e14327 (2010). 21179509 10.1371/journal.pone.0014327 PMC3002265

[b39] Xie G. D., Lu C. X., Leng Y. F., Zheng D. & Li S. C. Ecological assets valuation of the tibetan plateau. J. Nat. Resour. 18, 189–196 (in Chinese with English abstract) (2003).

[b40] Xie H., Zhou N. & Jian G. The construction and optimization of ecological networks based on natural heritage sites in Jiangsu Province. Acta Ecologica Sinica 34, 6692–6700 (in Chinese with English abstract) (2014).

[b41] Chang S. C., Tu C. J. & Chen H. Y. Ecological corridor in the urban area: case study in Kaohsiung City, Taiwan. Pract. Period. Hazard. Toxic Radioact. Waste Manage. 14, 76–88 (2010).

[b42] Gardner R. H. & Gustafson E. J. Simulating dispersal of reintroduced species within heterogeneous landscapes. Ecol. Modell. 171, 339–358 (2004).

[b43] Zeller K. A., Mcgarigal K. & Whiteley A. R. Estimating landscape resistance to movement: a review. Landscape Ecol. 27, 777–797 (2012).

[b44] Gonzalez J. R., Barrio G. D. & Duguy B. Assessing functional landscape connectivity for disturbance propagation on regional scales—A cost-surface model approach applied to surface fire spread. Ecol. Modell. 211, 121–141 (2008).

[b45] Bodin Ö. & Saura S. Ranking individual habitat patches as connectivity providers: Integrating network analysis and patch removal experiments. Ecol. Modell. 221, 2393–2405 (2010).

[b46] Pascual-Hortal L. & Saura S. Comparison and development of new graph-based connectivity indices: Towards the prioritization of habitat patches and corridors for conservation. Landscape Ecol. 35, 251–269 (2006).

[b47] Saura S. & Torné J. Conefor Sensinode 2.2: A software package for quantifying the importance of habitat patches for landscape connectivity. Environ. Modell. Softw. 24, 135–139 (2009).

[b48] Devi B. S., Murthy M. S. R., Debnath B. & Jha C. S. Forest patch connectivity diagnostics and prioritization using graph theory. Ecol. Modell. 251, 279–287 (2013).

[b49] Pereira M., Segurado P. & Neves N. Using spatial network structure in landscape management and planning: a case study with pond turtles. Landscape Urban Plan. 100, 67–76 (2011).

[b50] McRae B. H. & Kavanagh D. M. Linkage mapper connectivity analysis software. *Computer Software Program Produced by the Nature Conservancy in Seattle, WA, USA*. Available online: http://www.circuitscape.org/linkagemapper (accessed on 16 April 2016).

[b51] Saura S. & Pascual-Hortal L. A new habitat availability index to integrate connectivity in landscape conservation planning: comparison with existing indices and application to a case study. Landscape Urban Plan. 83, 91–103 (2007).

[b52] Fath B. D., Scharler U. M., Ulanowicz R. E. & Hannon B. Ecological network analysis: network construction. Ecol. Modell. 208, 49–55 (2007).

[b53] Ovaskainen O. Habitat specific movement parameters estimated using mark-recapture data and diffusion model. Ecology. 85, 242–257 (2004).

[b54] Saura S., Bodin Ö. & Fortin M. J. Editor’s Choice: Stepping stones are crucial for species’ long-distance dispersal and range expansion through habitat networks. J. Appl. Ecol. 51, 171–182 (2014).

